# Identification of acetylation-dependent regulatory mechanisms that govern the oncogenic functions of Skp2

**DOI:** 10.18632/oncotarget.740

**Published:** 2012-11-16

**Authors:** Zhiwei Wang, Hiroyuki Inuzuka, Jiateng Zhong, Pengda Liu, Fazlul H. Sarkar, Yi Sun, Wenyi Wei

**Affiliations:** ^1^ Department of Pathology, Beth Israel Deaconess Medical Center, Harvard Medical School, Boston, MA; ^2^ Department of Pathophysiology, Norman Bethune College of Medicine, Jilin University, Changchun, Jilin, P. R. China; ^3^ Department of Pathology and Oncology, Karmanos Cancer Institute, Wayne State University School of Medicine, Detroit, MI; ^4^ Division of Radiation and Cancer Biology, Department of Radiation Oncology, University of Michigan, Ann Arbor, MI

**Keywords:** acetylation, cancer, deacetylation, SIRT3, oncoprotein, ubiquitination, phosphorylation, F-box protein, Skp2, therapy

## Abstract

The Skp2 (S-phase kinase associated protein 2) oncoprotein is often highly expressed in various types of human cancers. However, the mechanistic basis of its oncogenic function, as well as the upstream regulatory pathway(s) that control Skp2 activities remains not fully understood. Recently, we reported that p300 acetylates Skp2 at two conserved lysine residues K68 and K71 within its NLS (Nuclear localization signal). This modification leads to increased Skp2 stability and cytoplasmic translocation, thus contributing to elevated Skp2 oncogenic potential. Moreover, we found that the SIRT3 tumor suppressor serves as the physiological deacetylase that antagonizes p300-mediated Skp2 acetylation. Furthermore, we showed that Skp2 governs E-cadherin ubiquitination and degradation in the cytosol. Consistent with this, we observed an inverse correlation between Skp2 and E-cadherin expression in clinical breast tumor samples. Therefore, our work elucidates a novel acetylation-dependent regulatory mechanism for Skp2 oncogenic functions.

## INTRODUCTION

**Figure 1:**
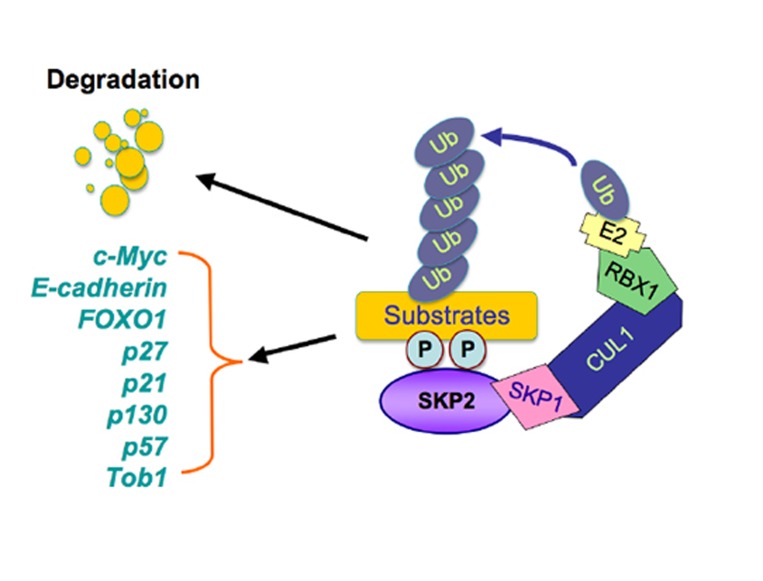
Illustrated pathway of Skp2-mediated degradation of its substrates The SCF (Skp1-Cullin 1-F-box) complex consists of four components: Skp1, Rbx1, Cullin1, and the F-box protein. While Skp2 recognizes its downstream substrates, cullin1-Rbx1 complex catalyzes the ubiquitin transfers from E2 to the substrates for targeted degradation by 26S-proteasome. The multiple substrates including p21, p27, p57, p130, Tob1, FOXO1, E-cadherin, and c-Myc have been identified.

The Skp2 F-box protein is a substrate recognizing component of the SCF (Skp1-Cullin 1-F-box) type of E3 ubiquitin-ligase complex [1]. It is known that the SCF complex consists of four crucial components including the invariable component Skp1, Rbx1 and Cullin1, and the interchangeable substrate-recruiting module, the F-box protein [1]. Among 70 putative F-box proteins, Skp2 is one of the best characterized F-box protein and has been shown to be involved in governing many cellular processes such as cell cycle regulation, cell proliferation, apoptosis, differentiation, and survival, in part through promoting the degradation of its substrate proteins [2, 3]. For example, Skp2 plays an important role in driving the cell cycle through the G1/S transition by promoting the destruction of the p27 tumor suppressor protein, an inhibitor of the CDK (Cyclin-dependent kinase) family of kinases [4, 5]. In addition to p27 [6], recent studies have identified numerous downstream Skp2 substrates including p21, p57, p130, c-Myc, FOXO1 (Forkhead box protein O1) [7-10], and Tob1[11] (Figure 1).

The fact that many Skp2 substrates are negative cell cycle regulators is consistent with the notion that Skp2 mainly functions as a proto-oncogene. Indeed, Skp2 has been found to be frequently overexpressed in a variety of human cancers including lymphomas [12], prostate cancer [13], melanoma [14], nasopharyngeal carcinoma [15], pancreatic cancer [16], and breast carcinomas [17, 18]. More importantly, in support of an oncogenic role for Skp2 in tumor progression, *Skp2^−/−^* mice has been found to be resistant to tumor development induced by loss of either the p53 or the PTEN tumor suppressor [19]. Although multiple signaling pathways such as phosphatidylinositol 3-kinase (PI3K)/Akt [20], AR (Androgen receptor) [21], PTEN (Phosphatase and tensin homolog) [13] and STAT1 (Signal transducers and activators of transcription) [22] have been reported to cross-talk with the Skp2 sinaling pathway and subsequently lead to tumorigenesis, the underlying mechanism(s) by which Skp2 is regulated *in vivo* remains largely elusive. Here, we will discuss the recent advances in our understanding of how Skp2 oncogenic role is governed *in vivo* by the novel acetylation-dependent mechanism, which is antagonized by the SIRT3 deacetylase.

### Skp2 is acetylated by p300

Multiple studies have shown that phosphorylation of Skp2 by Akt at Ser72 protects Skp2 from APC (Anaphase-promoting complex)/Cdh1-mediated proteolysis [23, 24]. However, Ser72 is only present in human and large mammals, but not conserved in mice, suggesting that Akt-mediated Skp2 phosphorylation might be a regulatory mechanism acquired late during the evolution. This implies that additional mechanisms might exist to regulate Skp2 activity. It is noteworthy that besides protein phosphorylation, protein acetylation has been recently demonstrated to emerge as another important type of post-translational modification that modulates many pathways involved in oncogenesis [25, 26]. More interestingly, while PI3K/Akt phosphorylates and activates acetyl-transferase p300 [27], Skp2 binds, but inhibits p300 to block p53-induced apoptosis [28]. Consistently, we found that interaction between p300 and Skp2 under both ectopic overexpression and endogenous co-immunoprecipitation conditions can readily be detected [29]. Furthermore, acetylation of Skp2 is detected using a specific acetyl-lysine antibody after ectopic expression of p300 [29]. Notably, we found that p300 acetylates the Skp2 oncoprotein at both K68 and K71 within its nuclear localization signal (NLS) region, just adjacent to the identified Ser72 Akt site [29]. Moreover, we found that p300-mediated Skp2 acetylation promotes Skp2 dimerization, suggesting that dimerization might affect the Skp2 substrate spectrum. To our knowledge, this is the first example demonstrating acetylation of an F-box protein, thereby suggesting the possibility of acetylation-dependent regulation of F-box protein(s) other than Skp2. In keeping with this note, recent large-scale mass spectrometry analyses have shown that a significant number of cellular proteins are acetylated [30, 31], although it is largely unclear how acetylation functions as a signaling mechanism to modulate downstream signaling and cellular physiology. Therefore, further studies are warranted to explore how analogous to phosphorylation-dependent regulation mechanism, acetylation could be utilized to govern the physiological functions of various F-box proteins.

Interestingly, Akt activates p300 acetyl-transferase activity to influence the Skp2 acetylation. However, p300 exerts its function independent of the Akt-Ser72-Skp2 pathway [29]. Therefore it is critical to further understand the possible redundancy or cross-talks between these two upstream regulatory pathways, Akt-mediated phosphorylation of Ser72 versus p300-mediated acetylation of Skp2, in terms of promoting Skp2 oncogenic signaling. It is possible that p300 and Akt are activated in response to distinct upstream signals to modulate Skp2 activity in specific settings. Alternatively, they share redundant functions with the p300 pathway being the ancestral mechanism of regulation, and Akt-mediated regulation acquired later in evolution. Obviously additional studies will be required to fully dissect the potential intercommunication between the p300 and Akt signal transduction pathways that modulate Skp2 activity.

### Skp2 is deacetylated by the SIRT3 tumor suppressor

The Sirtuin (SIRT) family of deacetylases have recently gained tremendous amount of attention due to their critical roles in many cellular functions [9]. The Sirtuins play important roles in a variety of cellular processes including aging, cellular metabolism and tumorigenesis [32]. Interestingly, Sirtuins are located in different cellular compartments, which may dictate the specific cellular function of each Sirtuin protein through promoting the deacetylation of various target proteins such as FOXO3a [33, 34], PPARγ (Peroxisome proliferators-activated receptor gamma) [35] and p53 [36]. For example, SIRT1, the best-characterized member of the mammalian Sirtuins, is located predominately in the nucleus [37, 38], whereas SIRT2 is found in the cytosol [39, 40]. SIRT3, SIRT4 and SIRT5, on the other hand, are localized mainly in the mitochondria [41, 42], [43].

Recent studies have also shown that SIRT3 has seemingly dichotomous role as either tumor promoter or tumor suppressor in cancer biology. For example, SIRT3 expression was found significantly higher in oral cancer cell lines and human oral cancer samples than in normal control [10]. In contrast, SIRT3 was reported to suppress tumor growth via induction of growth arrest and apoptosis in colorectal carcinoma, osteosarcoma cells, ovarian cancer, prostate cancer, suggesting that SIRT3 is tumor suppressor in these cancers [9]. Several published papers demonstrated a tumor suppressor role for SIRT3 via the ability of SIRT3 to negatively regulate ROS (reactive oxygen species) and HIF1-α (hypoxia inducible factor-1 α) [44, 45]. However, the exact mechanisms how SIRT3 is involved in cancer are largely unclear.

Our recent study showed that SIRT3 interacts with Skp2 and subsequently deacetylates Skp2 to suppress tumorigenesis [29]. Specifically, only SIRT3 and SIRT4 specifically interact with Skp2 among the various Sirtuin family members [29]. As SIRT3 is a tumor suppressor protein [45, 46], we further examined a potential role for SIRT3 in regulation of Skp2 acetylation. We detected a specific interaction between ectopically expressed as well as endogenous Skp2 and SIRT3. Moreover, depletion of SIRT3 caused an increase in endogenous Skp2 acetylation [29]. Additionally, depletion of SIRT3 also leads to a moderate increase in Skp2 abundance, which correlates with decreased expression of the Skp2 substrates p27 and p21. More importantly, the inverse correlation between Skp2 and SIRT3 immunohistochemical staining was observed in breast cancer tissues [29]. As SIRT3 has been implicated to possess tumor suppressor function [45, 46], this result suggests that loss of SIRT3 may lead to elevated Skp2 expression in breast cancers. In support of a tumor suppressor function for SIRT3, it has also been reported previously that *SIRT3^−/−^* cells displayed elevated ability to form tumors in a xenograft model and loss of SIRT3 has been identified as a frequent event in breast cancer cases [45, 46]. Using the xenograft model, we further showed that depletion of Skp2 retarded the *in vivo* tumorigenesis of *SIRT3^−/−^* cells [29]. Altogether, our results suggest that in our experimental settings, SIRT3 inhibited tumor growth mainly through deacetylation of Skp2 oncoprotein. Future study is directed to mechanistically understand how and whether SIRT3 exerts its tumor suppressor function by inactivating the Skp2 oncogenic pathway solely in a deacetylase-dependent manner.

### Skp2 acetylation governs its oncogenic function

The regulation of proteins by acetylation/deacetylation is considered as a significant post-translational regulatory mechanism to modify the specific enzyme's activity [47]. Hence, we intended to examine whether acetylation of Skp2 controls its oncogenic functions. Notably, we found that acetylation of Skp2 positively regulates its oncogenic activity partly through modulating its stability [29]. Moreover, acetylation of Skp2 exerts its function through promoting the destruction of its downstream targets such as p21 and FOXO1. To further support the role of Skp2 acetylation in tumorigenesis, depletion of endogenous SIRT3, which leads to increased acetylation of Skp2, promotes cell growth [29].

A canonical NLS was identified at the Skp2 amino terminus, and its function is highlighted by the fact that over-expression of an active Akt allele relocalizes a pool of Skp2 to the cytoplasm [23, 24]. Acetylation of lysines within an NLS has been reported to influence cellular localization [48-51]. Therefore, we detected whether acetylation of Skp2 NLS influences its localization. Consistent with the fact that Skp2 acetylation sites are found within its NLS, p300 promotes Skp2 cytoplasmic localization [29]. Interestingly, p300-induced Skp2 cellular localization is independent of Akt-mediated Skp2 phosphorylation [29]. In support of this notion, Skp2 cytoplasmic localization has been observed in many clinical tumor samples and is correlated with aggressive malignancy and poor diagnosis [17, 52-54]. Taken together, our results demonstrate that p300-mediated acetylation of Skp2 affects its stability and cytoplasmic localization, which in turn can influence its oncogenic activity. These results suggest that acetylation-mediated post-translational modifications might also control the function of other F-box proteins.

### Skp2 regulates cell migration through promoting E-cadherin destruction

It has been well accepted that enhanced cell migration is a critical phenotype required for cancer progression that leads to invasion and ultimately metastatic dissemination of tumor cells. In addition to controlling cell cycle progression, Skp2 has also been implicated in the regulation of cell migration. For example, a study showed that ectopic expression of Skp2 fused with an extra copy of an NES rescues the deficiency of cell motility in *Skp2*^−/−^ MEFs, in the absence of p27 degradation [23], indicating that cytoplasmic Skp2 may have a distinct function related to cell migration, independent of its major role in cell cycle regulation. Indeed, a correlation between elevated Skp2 protein expression and tumor metastasis has been noted in multiple tumors, including melanoma, lymphoma and breast carcinoma [55-57]. Furthermore, several studies have demonstrated that cytosolic Skp2 can positively regulate cell migration, although the molecular mechanisms are largely unknown [23]. As we have shown that acetylation promotes cytosolic localization, it became increasingly necessary to explore whether acetylation could influence Skp2-governed cellular migration.

To this end, E-cadherin is considered as a major player in EMT (epithelial to mesenchymal transition) [58]. It is accepted that after EMT, cells lose epithelial features including down-regulation of E-cadherin and gain mesenchymal characteristics such as upregulation of N-cadherin, fibronectin, and vimentin, leading to enhanced cell migration, invasion and metastasis [44, 59, 60]. Consistent with this notion, loss of E-cadherin is frequently observed in high-grade breast tumor samples [61-63]. However, it remains unclear whether enhanced proteolysis of E-cadherin also contributes to the reduced E-cadherin abundance in high-grade breast tumors. On the other hand, Skp2 is found to be frequently overexpressed in various types of cancers including breast cancer [1]. We therefore propose that aberrant Skp2 signaling may lead to elevated E-cadherin destruction that in turn profoundly affects cell migration and potentially EMT. In keeping with this hypothesis, our study indicates that Skp2 promotes cellular migration partly through promotion of E-cadherin destruction [29]. Furthermore, we observed an inverse correlation between Skp2 and E-cadherin expression in an array of breast cancer clinical samples [29]. Interestingly, we found that although ectopic expression of Skp2 in LNCaP cells leads to decreased expression of E-cadherin and α-E-catenin, other molecular markers for EMT were not significantly altered [29]. These results argue that cytoplasmic Skp2 preferentially promotes E-cadherin destruction to regulate cellular migration, but has nominal effects on other cell adhesion proteins, implying that additional oncogenic signaling, which is not currently fully underscored, is required for promoting full EMT. Therefore, additional studies are required to pinpoint these genetic alterations that might synergize with elevated Skp2 oncogenic signaling in facilitating cellular transformation and metastasis.

## CONCLUSION

**Figure 2:**
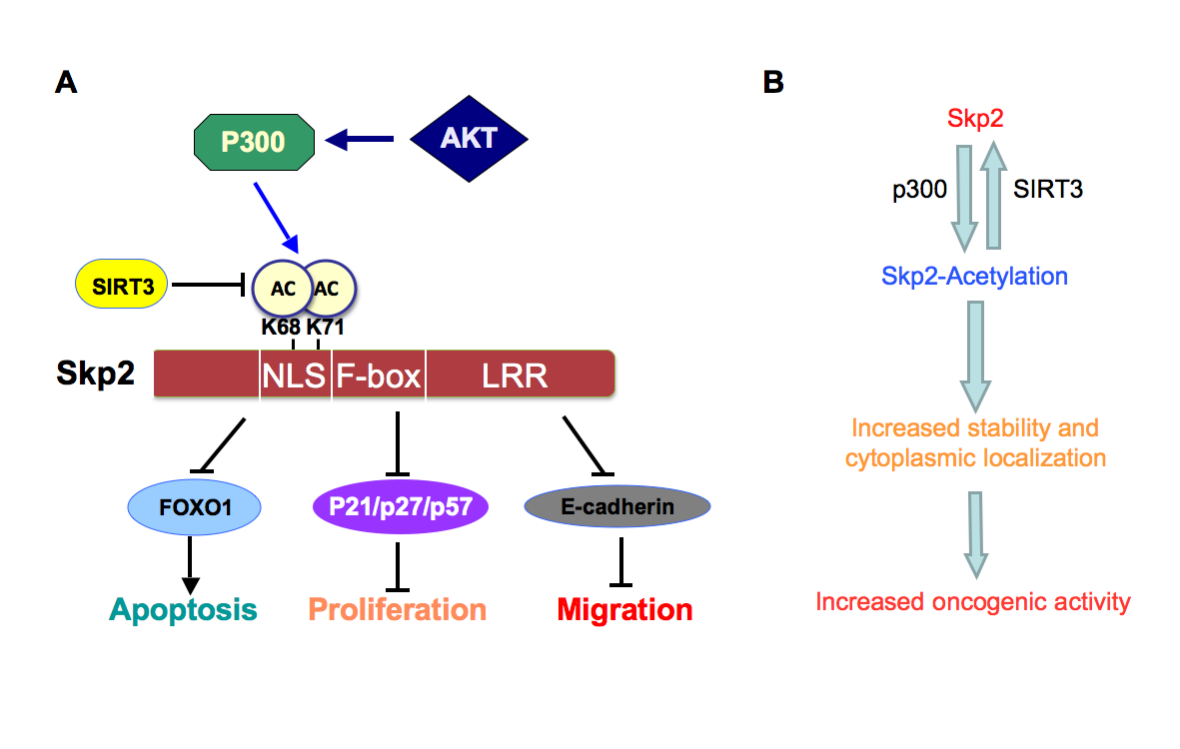
Proposed model for how Skp2 oncogenic role is regulated by p300 and SIRT3 A. Skp2 is acetylated by the p300 acetyl-transferase at both K68 and K71, leading to increased Skp2 stability and its oncogenic functions partly through promoting its cytoplasmic localization. Moreover, Skp2 promotes tumor cell migration via governing E-cadherin degradation. Furthermore, SIRT3 interacts with and deacetylates Skp2 to antagonize the acetylation by p300. B. Schematic illustration of how p300-dependent acetylation of Skp2, a process that can be antagonized by the SIRT3 deacetylase, leads to elevated Skp2 oncogenic functions in part by stabilizing Skp2 as well as promoting Skp2 cytoplasmic localization.

In conclusion, Skp2 plays an oncogenic role in the development and progression of human cancers through degradation of its downstream target proteins that control a variety of cellular processes such as cell proliferation, apoptosis, migration, invasion and metastasis. More importantly, we recently identified that Skp2 is acetylated by p300, resulting in its cytoplasmic localization and enhanced stability. We further demonstrate that the SIRT3 tumor suppressor interacts with and deacetylates Skp2 (Figure 2), suggesting that targeting Skp2 or the p300/SIRT3 axis could be a novel approach for the treatment of human cancers, especially those with up-regulation of Skp2. However, we recognize that although these studies provide the molecular basis for targeting Skp2 as novel anti-cancer therapeutic options, further in-depth studies are required to provide further insights to guide the design of effective therapeutics targeting Skp2 acetylation events to combat human cancers.
